# Antioxidant Effects of Some Drugs on Immobilization Stress Combined with Cold Restraint Stress

**DOI:** 10.3390/molecules14114505

**Published:** 2009-11-10

**Authors:** Mira Popovic, Snezana Janicijevic-Hudomal, Biljana Kaurinovic, Julijana Rasic, Svetlana Trivic, Matilda Vojnović

**Affiliations:** 1 Department of Chemistry, Faculty of Science, University of Novi Sad, Trg Dositeja Obradovica 3, 21000 Novi Sad, Serbia; 2 Institute of Pharmacology, Faculty of Medicine, University of Pristina (Kosovska Mitrovica), Serbia; 3 The Health Center, 21000 Novi Sad, Serbia

**Keywords:** immobilization stress, cold restraint stress, morphine, tramadol, bromocriptine, haloperidol, azithromycin, antioxidative status

## Abstract

The aim of this work was to investigate the effect on antioxidant potential of some commonly used drugs (morphine, tramadol, bromocriptine, haloperidol and azithromycin) on immobilization stress (IS) combined with cold restraint stress (CRS) in the rat. After the drug treatment the animals were kept immobilized in the cold chamber at 4±0.3ºC for 3 hours and then decapitaed and the livers were extracted. The following parameters were determined in the liver homogenate: content of reduced glutathione, activities of catalase, xanthine oxidase, glutathione reductase, glutathione peroxidase, peroxidase, and lipid peroxidation intensity. A battery of biochemical assays was used and the resulting data were statistically analyzed. Combined stress exhibited a prooxidative action (increased catalase activity, lowered content of reduced glutathione). Significantly enhanced catalase activity that was observed in all groups compared to the control indicates that the primary reactive oxygen species (ROS) metabolite is hydrogen peroxide, which decomposes very rapidly (very high catalase activity), thus hindering formation of OH radicals as the most toxic ROS. None of the tested drugs showed a protective effect on combined IS and CRS. The intensity of lipid peroxidation did not change either in the combined stress or under additional influence of the drugs. Probably, under our experimental conditions, the time was not sufficiently long to observe damage of lipid membrane by ROS.

## Introduction

Stress can be caused by various factors. It can come as a consequence of the action of biological factors (burns, injuries, surgical operation, hunger, high temperature variations, forced immobilization, severe metabolic disorders, etc.), but also psychophysical ones (fear, pain, sadness, etc) [1]. Every physical stress causes emotional stress and *vice versa*. Stress disturbs homeostasis and may induce various disorders. Every stress, whether physical or emotional, causes different response of the affected organism. Two different kinds of stress are surely to have additive effect. One of the causes of stress is also oxidative stress. Nowadays it is accepted that oxidative stress participates in the autogenesis of more than 100 diseases. Reactive oxygen species (ROS) formed in the oxidative stress react with biomolecules such as lipids, nucleic acid and proteins, changing their structure and thus their function, which leads to cell damage and even death. Opioids are the most potent analgesics used to treat acute cancer and non-cancer chronic pain [2,3,4]. Morphine is metabolized in the liver, gastrointestinal tract and kidneys [5,6].

Tramadol is an effective synthetic agent, centrally-acting analgesic, used for treating moderate to severe acute or chronic pain [7]. Like morphine, tramadol binds to μ**-**opioid receptors that are important for transmitting the sensation of pain throughout the body. 

Azithromycin (AZA) is a semisynthetic derivative of erythromycin which shows anti-inflammatory action towards acute inflammation. It binds within the peptide exit tunnel of the 50S ribosomal subunit, and thus inhibits bacterial protein synthesis [8]. Bakar *et al*. [9] showed that AZA exhibits antioxidant effects. This antibiotic with immunomodulatory effect, inhibits cytokine release, stimulates apoptosis, influences neutrophil function and mediates release, as well as inhibition of mucus secretion [10,11,12]. 

Bromocriptine (BRC), a dopamine (DA) D2 receptor agonist and mild D1 receptor antagonist, is widely used in the treatment of Parkinson’s disease since 1974, and in a broad spectrum of psychiatric disorders [13]. It reduces the formation of oxygen radicals in the course of normal metabolism of levodopa and DA [14]. Recent observations suggest that bromocriptine is an antioxidant that inhibits free radical formation and acts as a strong free radical scavenger *in vitro* and *in vivo* [15,16]. Bromocriptine protects cells by enhanced intrinsic defense mechanism against oxidative stress [17]. 

Haloperidol (HP), a commonly used drug for the treatment of schizophrenia, is metabolized by an oxidase, generates large quantities of oxyradicals and a potent toxic pyridinium-like metabolite [18]. This supports the suggestion that the treatment with typical neuroleptics can increase the oxyradicals as a result of possible increased drug and catecholamine metabolism and contribute to oxyradical injury in schizophrenic patients [19,20]. This neuroleptic drug is thought to exert its clinical effects through cerebral dopamine D2-receptors [21] and δ-opioid receptors [22,23]. It is known that a vast number of antioxidants (such as vitamin C, phenols, flavonoids, etc.) can, under certain conditions, show pro-oxidant activity. [24] Chronic treatment with neuroleptics increases free radical production and oxidative stress, and decreases the activity of antioxidant defense enzymes [25,26]. Haloperidol can cause oxidative stress under *some* conditions. However, this does not mean that it cannot act as an antioxidant under *some other* conditions. 

The commonly used medications—morphine, tramadol, bromocriptine, haloperidol and azythromycin have different chemical structures and different pharmacological actions. Because of the differences of their structure and function it was interesting to investigate whether they would show different or similar effects on measured biochemical parameters in the combined immobilization stress (IS) and cold restraint stress (CRS) [27,28,29]. 

Hence, the aim of this work was to investigate the effects of morphine, tramadol, bromocriptine, haloperidol, and azithromycin on antioxidative status of the liver in experimental animals that were exposed to IS and CRS. For this purpose we measured catalase (CAT, E.C. 1.11.1.6), peroxidase (Px, E.C. 1.11.1.7), glutathione peroxidase (GSHPx, E.C. 1.11.1.9), glutathione reductase (GSHR. E.C. 1.6.4.2) and xanthine oxidase (XOD, E.C. 1.2.3.2) activities, reduced glutathione (GSH) content, and lipid peroxidation (LPx) intensity, which are essential indicators of the oxidative stress [30].

## Results and Discussion

Table 1 shows the results of the action of tested drugs on the combined effect of IS and CRS on some biochemical parameters in rats. 

**Table 1:** Effects of drugs on combined IS and CRS.

Enzyme	OO group	IO group	IM group	IT group	IB group	IH group	IA group
XOD	8.33±0.84	1.91±0.19^c^	2.91±0.40^c,e^	3.33±0.10^c,f^	2.44±0.31^c,d^	2.84±0.36^c,e^	2.11±0.17^ c ^
CAT	4.42±0.29	14.40±0.55^c^	19.28±1.98^c,e^	16.29±0.45^c,f^	12.39±0.48^c,f^	24.20±1.33^c,f^	15.44±1.07^c^
Lpx	0.65±0.09	0.66±0.04	0.70±0.04	0.61±0.04	0.68±0.02	0.65±0.04	0.72±0.06
Px	11.36±1.91	10.58±0.95	10.97±0.88	11.73±1.20	11.08±1.71	17.22±1.51^b,f^	15.22±1.09^b,f^
GSH	2.69±0.28	0.53±0.09^c^	0.99±0.19^c,e ^	1.05±0.12^c,f^	0.52±0.08^c^	0.78±0.08^c,e^	1.18±0.41^c,d^
GSHPx	0.96±0.12	0.41±0.14^c^	0.58±0.04^c, d^	0.36±0.05^c^	0.35±0.07^c^	0.44±0.05^c^	0.38±0.08^c^
GSHR	2.93±0.20	4.02±0.33^c^	4.19±0.49^b^	3.42±0.10^b,e^	4.88±0.16^c,e^	5.09±0.76^c,d^	4.17±0.28^c ^

Activities of XOD, CAT, PX, GSHPx and GSHR are expressed in nmol/mg of protein min^-1^; Intensity of lipid peroxidation is expressed in nmol malondialdehyde/mg protein; Content of GSH is expressed in nmol GSH/mg protein n = 5; with respect to OO group: p > 0.05 (statistically nonsignificant), ^a ^p < 0.05, ^b ^p < 0.01, ^c ^p < 0.001 with respect to IO group: p > 0.05 (statistically nonsignificant), ^d ^p < 0.05 , ^e ^p < 0.01, ^f ^p < 0.001.

Statistical significance of differences was determined with respect to two control groups, OO and IO. The combination of IS and CRS caused statistically significant changes of the following parameters: it increased the activities of catalase and glutathione reductase and decreased activities of xanthine oxidase, glutathione peroxidase and content of reduced glutathione. Catalase activity was increased in all groups, compared to the OO control. Morphine, tramadol and haloperidol (IM, IT and IH groups) showed an increased and bromocriptine (IB group) a decreased activity of this enzyme activity with respect to the IO control, whereas the increase in the group IA was not statistically significant (Table 1).

Numerous studies have indicated that the exposure to cold leads to oxidative stress, that is the changes in the antioxidant enzyme and non-enzyme systems [27,28,29]. Results obtained for the investigated biochemical parameters in the IO group are probably a consequence of the combined IS and CRS. In the group IO there were no changes of the intensity of lipid peroxidation and activity of peroxidase compared to the group OO. Morphine and tramadol (IM and IT groups) did not essentially change the investigated parameters, that is they acted similarly as the combined stress itself (IO group). Of all the drugs tested only bromocriptine caused a statistically significant decrease of catalase and glutathione peroxidase activities, without lowering the content of reduced glutathione with respect to the IO group. Hence it comes out that this drug did not protect animals from the CRS. Haloperidol and azithromycin (groups IH and IA), in contrast to the combined stress itself and other applied drugs (groups IO, IM, IT and IB), increased the activity of peroxidase. It is essential to notice that the stress itself and the drugs applied changed no lipid peroxidation with respect to the control (OO group). The applied drugs exhibited very similar effects on the investigated parameters in comparison to the IO control. Azithromycin (group IA) and haloperidol (group IH) changed statistically significantly all investigated parameters compared to the OO group, an exception being the lipid peroxidation. Of all the drugs tested halperidol caused greatest changes of all the parameters (except for glutathione peroxidase activity) compared to the IO group. On the other hand, azithromycin caused a statistically significant change of only peroxidase activity and content of reduced GSH with respect to the IO group. It is important to emphasize that all the drugs with the exception of bromocriptine increased significantly the content of reduced glutathione compared to the IO group. 

Activity of xanthine oxidase was lowered in all groups compared to the OO group and increased (with the exception of IA group) with respect to IO group. 

Table 1 shows the results treated by Student's t test, to check whether there is a statistically significant difference between the measured parameters and controls (groups OO and IO). To test the total variability values and significance of differences observed between groups use was made of one-way ANOVA (one-way analysis of variance) and Tukey Snedecor test F and D values. 

Catalase activity was increased in all groups compared to OO control. Morphine, tramadol and haloperidol increased and bromocriptin decreased catalase activity compared to IO group (Table 1). This statistically significant difference was also confirmed by very highly significant difference between all groups with the exception of IA/IT (Table 2). 

Neither the IS combined with CRS nor the drugs tested changed the intensity of lipid peroxidation compared to the control OO group (Table 1). 

There were no statistically significant differences between groups in respect of lipid peroxidation in 12 combinations of groups. The lowest statistical significance of the difference between all the groups and for all tested parameters was observed in LPx (Table 2).

All the investigated parameters, treated by ANOVA test and the differences between the test groups, are presented in Table 2. As can be seen, in the majority of cases there is a highly significant difference between the measured biochemical parameters. 

**Table 2:** ANOVA test for measured biochemical parameters.

**XOD**	**00**	**I0**	**IM**	**IT**	**IB**	**IH**	**CAT**	**00**	**I0**	**IM**	**IT**	**IB**	**IH**
**I0**	6.42 +						**I0**	9.98 +					
**IM**	5.42 +	1.00 +					**IM**	14.85 +	4.88 +				
**IT**	4.99 +	1.42 +	0.43 +				**IT**	11.87 +	1.90 +	2.98 +			
**IB**	5.88 +	0.54 +	0.46 +	0.89 +			**IB**	7.97 +	2.01 +	6.89 +	3.91 +		
**IH**	5.49 +	0.93 +	0.07 -	0.49 +	0.40 +		**IH**	19.78 +	9.80 +	4.92 +	7.91 +	11.81 +	
**IA**	6.22 +	0.20 -	0.80 +	1.22 +	0.34 +	0.73 +	**IA**	11.01 +	1.04 +	3.84 +	0.86 -	3.05 +	8.76 +
**LPx**	**00**	**I0**	**IM**	**IT**	**IB**	**IH**	**Px**	**00**	**I0**	**IM**	**IT**	**IB**	**IH**
**I0**	0.01 -						**I0**	0.78 -					
**IM**	0.05 +	0.04 -					**IM**	0.40 -	0.38 -				
**IT**	0.04 -	0.05+	0.09 +				**IT**	0.37 -	1.15 +	0.76 -			
**IB**	0.03 -	0.02 -	0.02 -	0.07 +			**IB**	0.28 -	0.50 -	0.12 -	0.65 -		
**IH**	0.00 -	0.01 -	0.05 +	0.04 -	0.03 -		**IH**	5.86 +	6.64 +	6.26 +	5.49 +	6.14 +	
**IA**	0.08 +	0.07 +	0.03 -	0.12 +	0.04 -	0.08 +	**IA**	3.85 +	4.64 +	4.25 +	3.49 +	4.14 +	2.01 +
**GSH**	**00**	**I0**	**IM**	**IT**	**IB**	**IH**	**GSHPx**	**00**	**I0**	**IM**	**IT**	**IB**	**IH**
**I0**	2.16 +						**I0**	0.55 +					
**IM**	1.70 +	0.46 +					**IM**	0.38 +	0.16 +				
**IT**	1.64 +	0.52 +	0.05 -				**IT**	0.60 +	0.05 -	0.22 +			
**IB**	2.17 +	0.01 -	0.47 +	0.52 +			**IB**	0.61 +	0.06 +	0.23 +	0.01 -		
**IH**	1.91 +	0.25 +	0.21 +	0.27 +	0.25 +		**IH**	0.52 +	0.03 -	0.14 +	0.08 +	0.09 +	
**IA**	1.51 +	0.65 +	0.19 +	0.14 -	0.66 +	0.41 +	**IA**	0.58 +	0.04 -	0.20 +	0.01 -	0.03 -	0.06 -
**GSHR**	**00**	**I0**	**IM**	**IT**	**IB**	**IH**							
**I0**	1.09 +												
**IM**	1.25 +	0.17 -											
**IT**	0.49 +	0.60 +	0.76 +										
**IB**	1.95 +	0.86 +	0.69 +	1.46 +									
**IH**	2.15 +	1.07 +	0.90 +	1.66 +	0.21 -								
**IA**	1.24 +	0.15 -	0.02 -	0.75 +	0.71 +	0.92 +							

Results of the ANOVA test are represented for the differences between groups for the confidence level p<0.05; + statistically significant p < 0.05; - statistically nonsignificant p > 0.05; XOD: F = 118.48, p < 0.001; D = 0.34; CAT: F= 137.01, p < 0.001; D = 0.88; LPx: F = 2.10; p > 0.05; D = 0.04; Px: F = 14.01; p < 0.001; D = 1.13; GSH: F = 47.89; p < 0.001; D = 0.18; GSHPx: F = 31.56; p < 0.001; D = 0.06; GSHR: F = 14.70; p < 0.001; D = 0.33.

Lurie *et al.* [31] attributed the toxic effects of opioids on the cellular level to increased lipid peroxidation. Biological membranes contain large amounts of polyunsaturated fatty acids which readily react with ROS. Hence, lipid peroxidation can be used as a marker of the ROS-caused cell damages. A significant increase in lipid peroxidation was observed in rats that received an acute dose of cocaine [32]. Similarly, it has been found that lipid peroxidation is significantly increased in chronic heroin users [33]. In their work Atici *et al*. [34] demonstrated that treatment with morphine and tramadol yielded an increased MDA level, which suggests an increased lipid peroxidation [35]. It is interesting to notice that in our experiment the intentsity of lipid peroxidation changed neither by combined stress nor by the addional action of the drugs. We think that the time of exposure to the stress action (three hours) was not long enough to observe damages of lipid membranes by ROS. William *et al*. [36] showed that a decrease in the level of reduced glutathione was observed in the isolated rat hepatocytes in the case of incubation with different morphine concentration (yielding cell death), found that morphine acted on some antioxidant parameters of the liver: lowered content of reduced glutathione and activities of catalase, superoxide dismutase and glutathione peroxidase. Our findings are partly in agreement with theirs: the content of reduced glutathione was reduced as well as the glutathione peroxidase activity, but the catalase activity was markedly increased. A significantly increased catalase activity in all the groups compared to the control indicates that the primary metabolite of ROS is hydrogen peroxide, which decomposes very rapidly (very high catalase activity), hindering thus the formation of OH radical as the most toxic form of ROS. 

The investigated antioxidant parameters had very similar values for the groups IB and IO, which indicates that bromocriptine did not act protectively on IS combined with CRS. Lim *et al*. [17] showed that this drug enhanced intrinsic defense mechanism against oxidative stress. We think that under our experimental conditions the time of exposure to stress was not long enough to observe the protective effect of the drug [37]. 

The treatment with haloperidol (group IH) decreased the content of reduced glutathione and glutathione peroxidase activity, which is in correlation with the results of Azam [38]. Apart from the enzyme systems, the ROS detoxification involves also non-enzymatic antioxidants, a special role being played by the content of reduced glutathione. A decreased level of content of reduced glutathione and activities of superoxide dismutase and glutathione peroxidase in mice treated with haloperidol, Azam [38] explained by the degradation by free radicals during the detoxification process. 

The studies of Thaakur and Jyothi [20] and Azam [38] indicated that chronic treatment with haloperidol yielded decreased activities of catalase, superoxide dismutase, and glutathione peroxidase, increased intensity of lipid peroxidation and a decreased content of reduced glutathione. Treatment with haloperidol in our "acute" immobilization stress did not influence intensity of lipid peroxidation, while it decreased content of reduced glutathione and increased significantly activities of catalase and peroxidase. These differences in the results may be a consequence of the stress combination or the mode of treatment with haloperidol. Our results are not in correlation with the known fact that chronic treatment with haloperidol produces ROS [20,38]. Azithromycin in combination with IS combined with CRS exhibited an antioxidant effect, which is in agreement with the results obtained by Bakar *et al*. [9]. 

Further investigations and the determination of the other antioxidant parameters (superoxide dismutase, glutathione *S*-transferase, etc.) might contribute to a better understanding of the action of the combined IS and CRS in the presence of the mentioned drugs. A better understanding of the action of particular drugs on combined IS and CRS could also be gained by determining some other parameters such as increase in the catecholamine level, measurement of the levels of stress limiting factors such as endogeneous opioids, GABA, serotonin, acetylcholine, prostaglandine, etc. 

## 3. Experimental Section

### 3.1. General

This investigation was conducted on sexually mature male laboratory Wistar rats, with an average body weight of 200-230 g and aged up to three months. Rats were bred in the vivarium at the Center for Biomedical Investigation, Galenika a.d. Animals were kept in standard plexiglass cages at constant room temperature 22 ± 1 ºC, with circadian rhythm (day/night), and were fed standard laboratory rat feed, produced by the Veterinary Institute in Zemun. The number of rats was five per cage. Animals were treated according to the principles of the International Declaration Guide for Care and Use of Laboratory Animals (NIH publication № 85-23). 

### 3.2. Chemicals

Morphine hydrochloride and tramadol were obtained from Alkaloid AD (Skopje, FYR Macedonia); Bromocriptine was obtained from Zdravlje (Leskovac, Republic of Serbia); Haldol^®^ decanoate (haloperidol decanoate) was obtained from Janssen-Cilag, Division of Johnson & Johnson S.E. d.o.o. (Ljubljana, Slovenia); Hemomycin (azithromycin) was obtained from Hemofarm (Vrsac, Republic of Serbia). All chemicals used were of analytical grade.

### 3.3. Animal treatment

OO group – vivarium control – untreated animals; healthy untreated animals (animals that were not exposed to stress and were not treated with any drug) were used as a control group. 

IO group – combined IS and CRS without drug; IM group – morphine (10 mg/kg b.w.) in the initial stress phase 

IT group –tramadol (100 mg/kg b.w.) in the initial stress phase; IB group – bromocriptine 24 h before the stress (25 mg/kg b.w.), the next day 1.5 h prior to the IS (12.5 mg/kg b.w; IH group – haloperidol 30 min prior to the IS (25 mg/kg b.w.); IA group – azithromycin (250 mg/kg b.w.) during 5 days before the IS, and on the 5th day 2.5 h prior to the IS. 

The volume of drug solution applied intragastrically (nasogastric sond) did not exceed 1.0 mL, whereas the volume of intraperitoneally injected solution did not exceed 2.0 mL. 

Before the experiment, all animals were exposed to a 24-hour fasting period prior to treatment, but had free access to water, and were put each in a separate metabolic plexiglas cage with a wire floor to prevent coprofagia. Animals were returned to metabolic cages, but no water was given to them. 

The drugs were administered intragastrically (bromocriptine and azithromycin) or intraperiotoneally (morphine hydrochloride, tramadol and haloperidol). Average single doses of investigated drugs were selected on the basis of human dosage and Clark's formula.

Experiments were conducted in the same day interval (8-15 h). 

Combined IS and CRS test was performed by immobilizing animals in the cold chamber at 4 ± 0.3 ºC; the plexiglass cage volume was adjusted to the size of the animal, to restrain completely their movements. After keeping them in the immobilized chamber for three hours, the immobilized and drug treated animals (IO, IM, IT, IB, IH and IA groups) were sacrificed under ether anesthesia. 

The stress development was monitored under the influence of different groups of drugs, namely, opiate agonists and antagonists, dopamine agonists and antagonists, antibiotics and nonsteroidal antiinflammatory drugs.

### 3.4. Biochemical assays

Animals after treatments were decapitated and the livers were extracted. The liver was homogenized in a Potter homogenizer with 50 mM TRIS-HCl sucrose pH 7.40 in a ratio 1:3 at 4 °C. The obtained homogenate was filtered and the following biochemical parameters were determined: extent of lipid peroxidation LPx was determined after Buege and Aust [39]; activities of peroxidase (Px) were measured after Simon *et al*. [40]; catalase (CAT) after Beers and Sizer [41]; glutathione peroxidase (GSHPx) after Chin *et al*. [42]; xanthine oxidase (XOD) after Bergmayer [43]; glutathione reductase (GSHR) after Glatzle and Vuillenmir [44]; content of reduced glutathione (GSH) after Kapetanović and Mieyal [45], and content of protein after Gornall *et al*. [46].

### 3.5. Statistical analysis

Results of biochemical analyses are presented as the mean value ± standard deviation. The differences between control and test groups were analyzed using the Student t-test (significant difference at p ≤ 0.05 confidence level). Using one-way ANOVA and Tukey Snedecor test F and D values, parameters which present the total variability values and significance of differences observed between groups, were assessed.

## 4. Conclusions

The combination of IS and CRS (IO group) significantly decreased xanthine oxidase activity and increased activity of catalase. As for the reduced GSH cycle, content of reduced glutathione and glutathione peroxidase activity, they were decreased, whereas activity of glutathione reductase was increased with respect to the OO group. In all groups under combined stress and drugs, xanthine oxidase activity was significantly lowered, compared to the OO group. The drugs in combination with stress did not change essentially measured parameters with respect to OO group. Combination of the stress with haloperidol and azithromycin caused a statistically significant increase of peroxidase activity compared to both controls (OO and IO groups). The combination of IS and CRS with the other drugs did not affect peroxidase activity. The intensity of lipid peroxidation did not change either in the combined stress or under additional influence of the drugs. Probably, under the conditions of our experiment, the time was not sufficiently long to observe damage of lipid membrane by reactive oxygen species (ROS). Significantly enhanced catalase activity that was observed in all groups compared to the control indicates that the primary ROS metabolite is hydrogen peroxide, which decomposes very rapidly (very high catalase activity), hindering thus formation of OH radicals as the most toxic ROS. None of the tested drugs showed a protective effect on combined IS and CRS. Cold immobilization stress is an extreme condition and it cannot be carried out on people, although results obtained on animals could indicate possible human reactions to it. Thus, we may assume that the same drug effects might operate in humans. 
